# Butyrate Prevents Induction of CXCL10 and Non-Canonical IRF9 Expression by Activated Human Intestinal Epithelial Cells via HDAC Inhibition

**DOI:** 10.3390/ijms23073980

**Published:** 2022-04-02

**Authors:** Sandra G. P. J. Korsten, Laura Peracic, Luka M. B. van Groeningen, Mara A. P. Diks, Herman Vromans, Johan Garssen, Linette E. M. Willemsen

**Affiliations:** 1 Division of Pharmacology, Utrecht Institute for Pharmaceutical Sciences, Utrecht University, 3584 CG Utrecht, The Netherlands; l.peracic@students.uu.nl (L.P.); l.m.b.vangroeningen@students.uu.nl (L.M.B.v.G.); m.a.p.diks@uu.nl (M.A.P.D.); j.garssen@uu.nl (J.G.); 2 Tiofarma B.V., 3261 ME Oud-Beijerland, The Netherlands; h.vromans@uu.nl; 3 Division of Pharmaceutics, Utrecht Institute for Pharmaceutical Sciences, Utrecht University, 3584 CG Utrecht, The Netherlands; 4 Nutricia Research B.V., 3584 CT Utrecht, The Netherlands

**Keywords:** intestinal epithelial cells, short chain fatty acids, CXCL10, STAT1 pathway

## Abstract

Non-communicable diseases are increasing and have an underlying low-grade inflammation in common, which may affect gut health. To maintain intestinal homeostasis, unwanted epithelial activation needs to be avoided. This study compared the efficacy of butyrate, propionate and acetate to suppress IFN-γ+/−TNF-α induced intestinal epithelial activation in association with their HDAC inhibitory capacity, while studying the canonical and non-canonical STAT1 pathway. HT-29 were activated with IFN-γ+/−TNF-α and treated with short chain fatty acids (SCFAs) or histone deacetylase (HDAC) inhibitors. CXCL10 release and protein and mRNA expression of proteins involved in the STAT1 pathway were determined. All SCFAs dose-dependently inhibited CXCL10 release of the cells after activation with IFN-γ or IFN-γ+TNF-α. Butyrate was the most effective, completely preventing CXCL10 induction. Butyrate did not affect phosphorylated STAT1, nor phosphorylated NFκB p65, but inhibited IRF9 and phosphorylated JAK2 protein expression in activated cells. Additionally, butyrate inhibited *CXCL10*, *SOCS1*, *JAK2* and *IRF9* mRNA in activated cells. The effect of butyrate was mimicked by class I HDAC inhibitors and a general HDAC inhibitor Trichostatin A. Butyrate is the most potent inhibitor of CXCL10 release compared to other SCFAs and acts via HDAC inhibition. This causes downregulation of *CXCL10*, *JAK2* and *IRF9* genes, resulting in a decreased IRF9 protein expression which inhibits the non-canonical pathway and CXCL10 transcription.

## 1. Introduction

The increase in the number of non-communicable diseases among humans is one of the major global health challenges of this century. A high-risk factor for non-communicable diseases is the consumption of a Western diet consisting of high fat and low fibers. Such a diet can result in changes in microbiome diversity, leading to dysbiosis [1,2]. Dysbiosis is associated with a disrupted intestinal barrier, leading to increased systemic endotoxin levels and low-grade inflammation both locally in the gut and systemically [3,4,5,6,7]. Low-grade inflammation causes neurological, metabolic and other subsequent immunological changes which are involved in the development of non-communicable diseases, such as cardiovascular diseases, diabetes, inflammatory diseases, asthma, allergies and even cancer. The development of these diseases may relate to the insufficient intake of fermentable fibers which serve as a substrate for the gut microbiota and are converted into short chain fatty acids (SCFAs), such as butyrate, propionate and acetate. These SCFAs improve intestinal barrier function [8,9,10,11,12,13] and have anti-inflammatory properties [5,11,14,15,16,17,18,19,20]. Preclinical studies have shown that reduced levels of SCFAs can result in intestinal barrier disruption and local inflammation, causing the passage of endotoxins over the intestinal barrier, leading to systemic low-grade inflammation [21,22]. The intestinal barrier and local systemic immune homeostasis may be improved by supplementation with SCFAs. However, more insight is required regarding the mechanisms by which SCFAs act to protect against unwanted inflammatory responses in mucosal tissues via their impact on the intestinal epithelial cells (IECs). 

The intestinal barrier is disrupted and the intestinal epithelium is activated by several pro-inflammatory cytokines, including interferon-gamma (IFN-γ) and tumor necrosis factor-alpha (TNF-α) [23,24,25,26]. IFN-γ activates the signal transducer and activator of transcription 1 (STAT1) signaling cascade upon binding to its receptor and signals via the canonical or non-canonical cascade [27]. In the canonical cascade, the dimerized IFN-γ-receptor is activated by phosphorylation of Janus kinase 1 (JAK1) and Janus kinase 2 (JAK2) domains which downstream facilitates dimerization of intracellular STAT1 monomers. The STAT1 dimer is phosphorylated and travels into the nucleus to act as a transcription factor via gamma-activated sequences, resulting in the expression of target genes, such as C-X-C motif chemokine ligand 10 (CXCL10). In the non-canonical cascade, the STAT1 dimer will not phosphorylate but bind to interferon regulatory factor 9 (IRF9). This complex will travel to the nucleus to act as a transcription factor via interferon-stimulated response elements, also resulting in the expression of target genes, such as CXCL10 [28]. In both activated cascades, suppressors of cytokine signaling 1 (SOCS1) can directly inhibit JAK1 and JAK2, ensuring a negative feedback mechanism [29]. The activation of these cascades and release of pro-inflammatory CXCL10 will accelerate Th-1 type inflammatory responses [30]. Blockage of CXCL10 release reduces this Th-1 type response, reduces inflammation and, in turn, could be beneficial in maintaining immune homeostasis and intestinal barrier integrity. 

Another common pro-inflammatory mediator, TNF-α, activates the nuclear factor kappa-light-chain-enhancer of activated B cells (NFκB) pathway which, amongst others, will result in the release of pro-inflammatory C-X-C motif chemokine ligand 8 (CXCL8). Moreover, TNF-α is known to intensify IFN-γ-induced epithelial activation, resulting in a synergetic effect on CXCL10 release [31]. This synergy can be explained by either an upregulation of the IFN-γ-receptor by TNF-α [32,33], an increased IFN-γ induced STAT1 phosphorylation by TNF-α [33,34,35] or the enhanced recruitment of the STAT1 dimer to the promotor site by TNF-α [33].

SCFAs are known inhibitors of CXCL10 release and the STAT1 signaling cascade [36,37,38,39,40,41]. The effect of SCFA butyrate has already been studied in IFN-γ-activated intestinal epithelial cells; however, it is unknown whether propionate and acetate are capable of inhibiting IFN-γ-mediated signal transduction in intestinal epithelial cells as well. In addition, it is unknown whether SCFAs are still effective in inhibiting CXCL10 release when IECs are exposed to both IFN-γ and TNF-α instead of IFN-γ alone. Research related to how butyrate may affect CXCL10 release mainly focusses on the canonical STAT1 signaling cascade [37,39]. Whether butyrate affects CXCL10 release via the non-canonical STAT1 signaling cascade is largely unknown.

SCFAs inhibit histone deacetylase (HDAC) [15]. HDAC can be subdivided into class I (HDAC1, HDAC2, HDAC3 and HDAC8), class IIa (HDAC4, HDAC5, HDAC7 and HDAC9), class IIb (HDAC6 and HDAC10) and class IV (HDAC11) [42]. HDAC inhibitors inhibit histone deacetylase and can regulate gene expression. Butyrate is a potent class I and class IIa HDAC inhibitor [43]. Propionate is a similar or slightly less potent HDAC inhibitor [44,45] while the effects of acetate are unclear, with studies reporting only an effect in high concentrations or no effect at all [45,46,47]. It is known that a nonspecific HDAC inhibitor reduces CXCL10 release, and that HDAC is of importance for gut homeostasis and can interact with the canonical and non-canonical STAT1 signaling cascade [38,48,49,50,51,52,53]. It is unknown whether the HDAC inhibitory properties of butyrate are strong enough to affect these signaling cascades in a similar way and whether more specific HDAC inhibitors are capable of reducing CXCL10 release.

In this study, we compared the efficacy of butyrate, propionate or acetate to suppress IFN-γ and IFN-γ+TNF-α-induced intestinal epithelial activation in association with their capacity to inhibit HDAC, while studying the canonical and non-canonical STAT1 signaling cascade. In addition, we will investigate the effect of more specific HDAC inhibitors on CXCL10 release to better understand which HDACs are involved in the effect of SCFAs. We hypothesize that SCFAs, and in particular butyrate, affect the STAT1 signaling cascade and CXCL10 release via HDAC inhibition.

## 2. Results

### 2.1. SCFAs Prevent the Release of CXCL10 by Activated IECs

**Figure 1:**
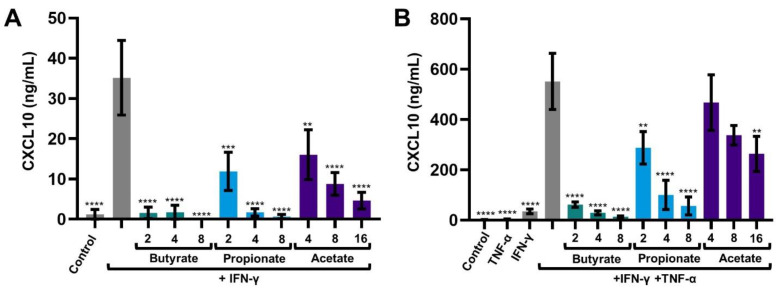
CXCL10 release by intestinal epithelial cells after 24 h, activated with IFN-γ (**A**) or IFN-γ+TNF-α (**B**) and treated with 2, 4, 8 and 16 mM butyrate, propionate or acetate. Data are represented as mean ± SEM (*n* = 3). Significant differences are shown as ** *p* < 0.01, *** *p* < 0.001, **** *p* < 0.001 compared to IFN-γ (**A**) or IFN-γ+TNF-α (**B**) activated cells.

IECs were treated with butyrate, propionate or acetate and activated with IFN-γ or IFN-γ+TNF-α. CXCL10 and CXCL8 were measured in the supernatant collected after 1, 4, 16 and/or 24 h. After 24 h CXCL10 release of IFN-γ-activated cells was increased compared to medium control. CXCL10 release of combined IFN-γ+TNF-α-activated cells was increased by almost 15-fold compared to IFN-γ-activated cells. CXCL10 release of IFNγ and IFNγ+TNFα-activated cells was inhibited by all SCFAs in a dose-dependent manner. Acetate was the least effective, followed by propionate and butyrate. Butyrate was most effective, and completely prevented the induced CXCL10 release at a dose of only 2 mM, not only of IFN-γ-activated cells but of IFN-γ+TNF-α-activated cells as well (Figure 1A,B).

**Figure 2:**
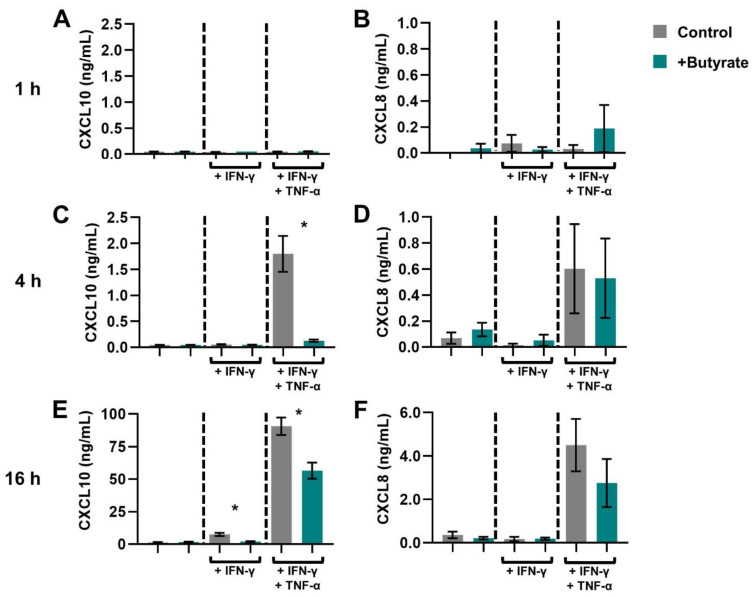
Chemokine release in time by intestinal epithelial cells activated with IFN-γ or IFN-γ+TNF-α and treated with 2 mM butyrate (green bars). CXCL10 (**A**,**C**,**E**) and CXCL8 (**B**,**D**,**F**) release was measured after 1, 4 and 16 h incubation. Data are represented as mean ± SEM (*n* = 4). Significant differences are shown as * *p* < 0.05 tested with a paired *t*-test between control and butyrate treated cells.

The kinetics of the release of CXCL10 and CXCL8 was studied and the inhibitory effect of butyrate was further investigated. CXCL10 was detected after 4 h of IFN-γ+TNF-α exposure and after 16 h of exposure to IFN-γ alone. An amount of 2 mM Butyrate reduced CXCL10 at 4 h in IFN-γ+TNF-α-activated IECs and at 16 h in IFN-γ and IFN-γ+TNF-α-activated IECs (Figure 2A,C,E). IFN-γ+TNF-α-activated cells additionally released CXCL8, which was not affected by 2 mM butyrate (Figure 2B,D,F). None of the treatments affected the viability of the cells (Appendix A).

### 2.2. Butyrate Reduces Proteins Related to the Non-Canonical STAT1 Signaling Cascade

**Figure 3:**
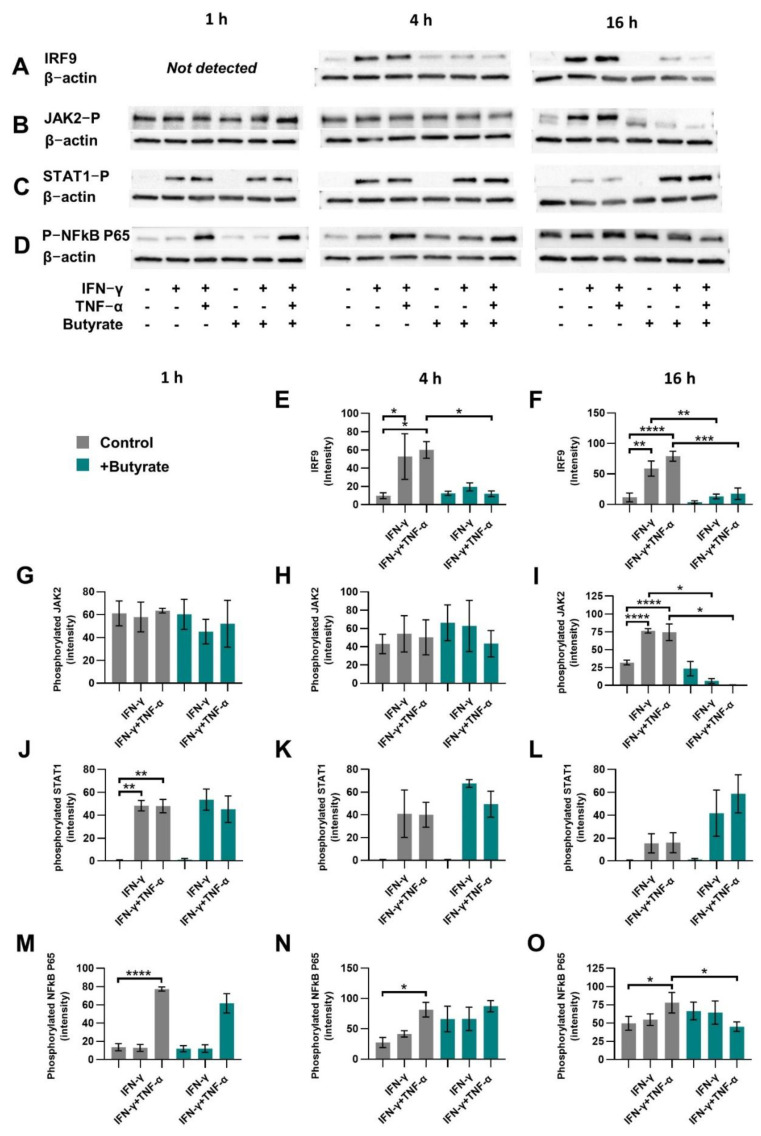
Canonical or non-canonical STAT1 pathway protein expression. Cells were activated with IFN-γ or IFN-γ+TNF-α and treated with 2 mM butyrate (green bars) for 1 h, 4 h or 16 h. IRF9 (**A**,**E**,**F**), phosphorylated JAK2 (**B**,**G**–**I**), phosphorylated STAT1 (**C**,**J**–**L**) and phosphorylated NFkB p65 (**D**,**M**–**O**) protein expression was determined. Representable blots of proteins of interest and β-actin control are shown in A-D. Data are represented as mean ± SEM (*n* = 3). Significant differences are shown as * *p* < 0.05, ** *p* < 0.01, *** *p* < 0.001, **** *p* < 0.001, medium control as compared to 2 mM butyrate control or to IFN-γ or IFN-γ+TNF-α-activated intestinal epithelial cells in absence of butyrate. Activated cells were also compared to activated cells treated with butyrate.

IECs were activated with IFN-γ or IFN-γ+TNF-α and treated with 2 mM butyrate because this concentration showed to be effective in the prevention of induced CXCL10 release. After 1, 4 and 16 h, cells were lysed and IRF9, phosphorylated JAK2, phosphorylated STAT1 and phosphorylated NFκB P65 protein levels were quantified; β-actin served as a control for equal protein loading (Figure 3A–D). 

IRF9 expression increased in IFN-γ and IFN-γ+TNF-α-activated cells at 4 h and 16 h of incubation, while at 1 h, IRF9 could not be detected. A quantity of 2 mM butyrate inhibited IRF9 induction at 4 and 16 h, maintaining similar levels as for the control cells (Figure 3A,E,F). 

Phosphorylated JAK2 increased in IFN-γ and IFN-γ+TNF-α-activated cells at 16 h of incubation and 2 mM butyrate decreased JAK2 phosphorylation at this timepoint (Figure 3B,G–I). 

Phosphorylated STAT1 increased in IFN-γ and IFN-γ+TNF-α-activated cells at 1 h of incubation and 2 mM butyrate did not affect this (Figure 3C,J–L). 

Phosphorylated NFκB P65 increased only in IFN-γ+TNF-α-activated cells at 1, 4 and 16 h of incubation, and 2 mM butyrate inhibited this only at 16 h of incubation (Figure 3D,M–O).

### 2.3. SCFAs Inhibit HDAC Activity in HT-29

**Figure 4:**
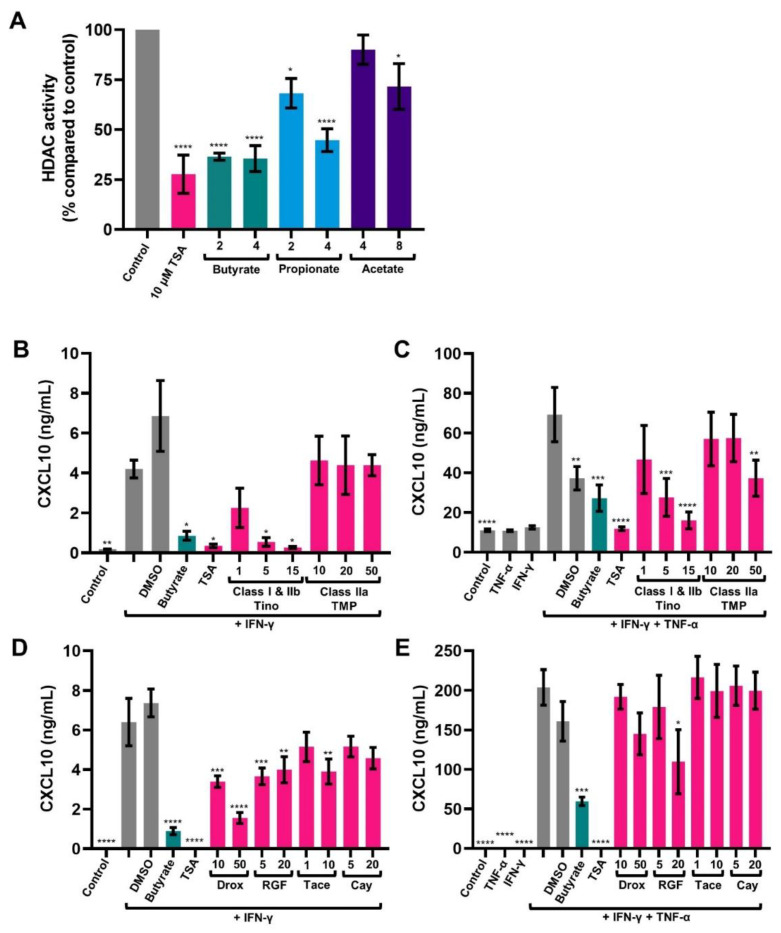
The effect short chain fatty acids on histone deacetylase (HDAC) activity (**A**) and the effect of HDAC inhibitors on CXCL10 release (**B**–**E**). HDAC activity of IECs after 24 h exposure to butyrate (2, 4 mM), propionate (4, 8 mM), acetate (4, 8 mM) or Trichostatin A (TSA) (10 μM) (**A**). Data are represented as mean ± SEM (*n* = 3). Significant differences are shown as * *p* < 0.05, **** *p* < 0.001 compared to control. CXCL10 release by intestinal epithelial cells after 24 h, activated with IFN-γ (**B**,**C**) or IFN-γ+TNF-α (**D**,**E**) and treated with 2 mM butyrate; 10 μM TSA; 1, 5, 15 μM tinostamustine (Tino); 10, 20, 50 μM TMP269 (TMP) (**B**,**C**) or with 2 mM butyrate; 10 μM TSA; 10, 50 μM droxinostat (Drox); 5, 20 μM RGFP966 (RGF); 1, 10 μM tacedinaline (Tace); 5, 20 μM Cay10683 (Cay) (**D**,**E**). 0.5% DMSO served as a control. Data are represented as mean ± SEM (*n* = 3). Significant differences are shown as * *p* < 0.05, ** *p* < 0.01, *** *p* < 0.001, **** *p* < 0.001 compared to IFN-γ-activated cells (**B**,**D**) or IFN-γ+TNF-α-activated cells (**C**,**E**).

SCFAs, and in particular butyrate, are known HDAC inhibitors [54]. Here, their inhibitory capacity was studied in IECs HT-29. All SCFAs reduced HDAC activity compared to medium control. Acetate was the least effective, followed by propionate and butyrate. Quantities of 2 and 4 mM butyrate reduced HDAC activity similar to 10 μM Trichostatin A (TSA), a general HDAC inhibitor (Figure 4A). 

### 2.4. HDAC Inhibitors Mimic the Effect of Butyrate in Activated IECs

It was investigated whether HDAC inhibitors mimic the anti-inflammatory effects of butyrate. Again, CXCL10 release of IFN-γ-activated cells after 24 h was increased compared to medium control. CXCL10 release of combined IFN-γ+TNF-α-activated cells was increased by almost 10-fold compared to IFN-γ-activated cells (Figure 4B–E). CXCL10 release of IFN-γ or IFN-γ+TNF-α-activated IECs was inhibited by general HDAC inhibitor TSA and a higher concentration of the class I and IIb HDAC inhibitor tinostamustine. In contrast, CXCL10 release was not inhibited by the class IIa HDAC inhibitor TMP269. Except for IFN-γ+TNF-α-activated cells treated with 50 μM TMP269, which showed a significant reduction in CXCL10 release, as well as the DMSO control (Figure 4B,C). HDAC class I consists of HDAC 1, 2, 3, 8 and class IIb consists of HDAC 6 and 10. The more specific HDAC inhibitors droxinostat (which inhibits HDAC 3, 6 and 8), RGFP966 (which inhibits HDAC 3 and to a lesser extent 1 and 2) and tacedinaline (which inhibit HDAC 1, 2 and 3) inhibited CXCL10 release of IFN-γ-activated cells, but only 20 μM of RGFP966 inhibited CXCL10 release of IFN-γ+TNF-α-activated (Figure 4C,D). Cay10683 (which inhibits HDAC 2 and 6) did not inhibit CXCL10 release of activated IECs.

### 2.5. Butyrate Inhibits CXCL10 Transcription in Activated IECs

HDAC and HDAC inhibitors affect gene transcription; therefore the mRNA expression of *CXCL10*, *IRF9*, *JAK2* and *SOCS1* genes in IFN-γ or IFN-γ+TNF-α-activated cells were studied at 4 h incubation with 2 mM butyrate or 10 μM TSA. *RPS-13* served as a household gene.

**Figure 5:**
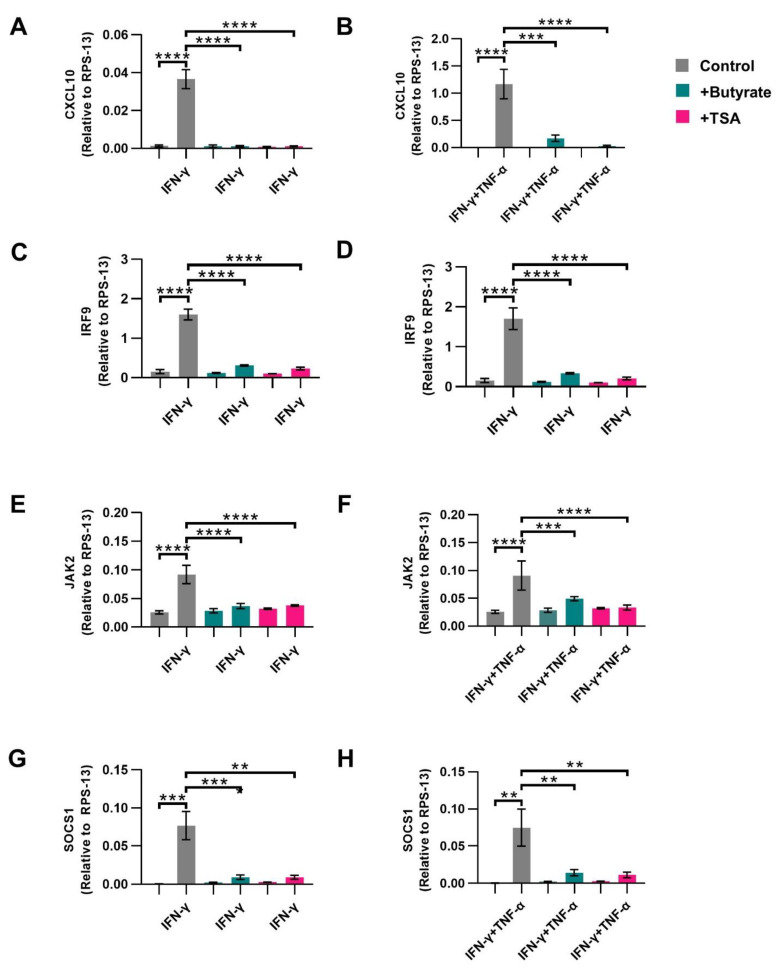
The effect of butyrate and histone deacetylase (HDAC) inhibitor Trichostatin A (TSA) on mRNA expression of genes related to the STAT1 signaling cascade. Intestinal epithelial cells were activated with IFN-γ or IFN-γ+TNF-α and treated with 2 mM butyrate (green bars) or 10 μM TSA (pink bars) for 4 h. mRNA expression was measured in *CXCL10* (**A**,**B**), *IRF9* (**C**,**D**) *JAK2* (**E**,**F**) and *SOCS1* (**G**,**H**). Data are represented as mean ± SEM (*n* = 4). Significant differences are shown as ** *p* < 0.01, *** *p* < 0.001, **** *p* < 0.001 Control compared to butyrate, TSA, IFN-γ (**A**,**C**,**E**,**G**) or IFNγ+TNFα (**B**,**D**,**F**,**H**) activated cells and activated cells compared to butyrate or TSA treated activated cells.

*CXCL10* (Figure 5A,B), *IRF9* (Figure 5C,D), *JAK2* (Figure 5E,F) and *SOCS1* (Figure 5G,H) gene expression was increased in IFN-γ and IFN-γ+TNF-α-activated cells, which was completely prevented by both butyrate and TSA (Figure 5A–F). 

### 2.6. HDAC Inhibitor TSA Prevents Induced IRF9 Expression Similar to Butyrate

**Figure 6:**
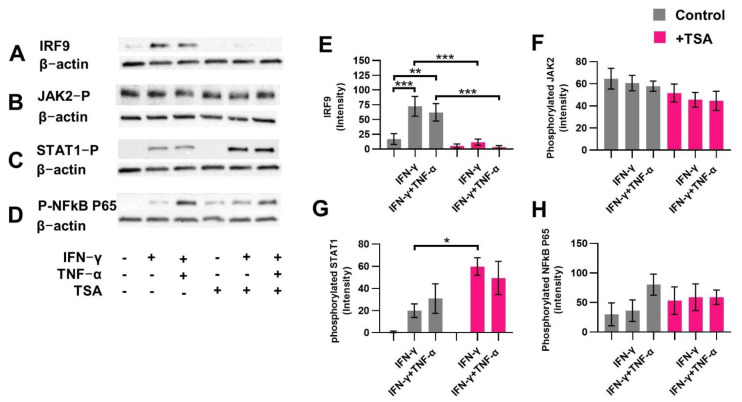
The effect of histone deacetylase inhibitor Trichostatin A (TSA) on downstream proteins of the STAT1 signaling cascade. Protein expression in IFN-γ or IFN-γ+TNF-α-activated intestinal epithelial cells (IECs) after 4 h incubation with 10 μM TSA (pink bars). Proteins measured were IRF9 (**A**,**E**), phosphorylated JAK2 (**B**,**F**), phosphorylated STAT1 (**C**,**G**), phosphorylated NFκB p65 (**D**,**H**). Data are represented as mean ± SEM (*n* = 3). Significant differences are shown as * *p* < 0.05, ** *p* < 0.01, *** *p* < 0.001, control compared to IFN-γ-activated IECs, IFN-γ+TNF-α-activated IECs or 10 μM TSA control. Activated cells were compared to activated cells treated with TSA.

To investigate whether HDAC inhibitors affect the STAT1 signaling cascade similarly to butyrate, protein expression and phosphorylation of proteins related to the cascade were determined. Therefore, cells were activated with IFN-γ or IFN-γ+TNF-α and treated with 10 μM TSA. TSA prevented the induction of IRF9 in IFN-γ and IFN-γ+TNF-α-activated cells (Figure 6A,E) and did not affect the expression of phosphorylated JAK2 (Figure 6B,F) and phosphorylated NFκB P65 (Figure 6D,H). TSA significantly induced phosphorylation of STAT1 only in IFN-γ-activated cells (Figure 6C,G).

## 3. Discussion

SCFAs are known to improve intestinal barrier function and to act anti-inflammatory. In the present study, IFN-γ-activated IECs in the presence or absence of TNF-α were used as a model to compare the efficacy of SCFAs in inhibiting CXCL10 release. In addition, their capacity to affect the canonical and non-canonical STAT1 signaling cascade was studied while investigating the role of HDAC inhibition. 

SCFAs, and most prominently butyrate, completely prevented the cytokine-induced CXCL10 release, not only of IFN-γ-activated IECs but also of IFN-γ+TNF-α-activated IECs. Importantly, IFN-γ+TNF-α induced CXCL10 release up to 10–15-fold higher concentrations when compared to IFN-γ-activation alone. The synergy between these two pro-inflammatory cytokines, IFN-γ and TNF-α, is a known phenomenon with different proposed underlying mechanisms [31,32,33,35,53]. The inhibitory effect of butyrate on the CXCL10 release induced by IFN-γ+TNF-α to our knowledge has not been studied before and indicates the strong anti-inflammatory efficacy of butyrate. Acetate was least effective in preventing CXCL10 release, while butyrate completely blocked CXCL10 release of both IFN-γ and IFN-γ+TNF-α-activated cells at a concentration of only 2 mM. These findings are in line with previous research where CXCL10 release was reduced in mature dendritic cells, LPS stimulated whole blood and IFN-γ-stimulated human colonic subepithelial myofibroblasts, after treatment with butyrate or propionate, but not after treatment with acetate [38,40,41]. TNF-α is known to induce CXCL8 release by IECs via NFκB signaling. Even though butyrate is known as an NFκB inhibitor [55,56], 2 mM butyrate did not affect CXCL8 release at 1, 4 and 16 h of incubation, nor did it affect TNF-α-induced phosphorylated NFκB p65 at 1 and 4 h of incubation. Therefore, butyrate did not block TNF-α-induced epithelial activation at these early time points, and thus the selective preventive effect of butyrate on synergistically induced CXCL10 release by IFN-γ+TNF-α-activated IECs could not be explained by the early blockage of the NFκB signaling cascade. However, butyrate did inhibit NFκB p65 phosphorylation at 16 h of incubation. In other studies, butyrate was found to inhibit CXCL8 release of endothelial and intestinal epithelial cells after pretreatment for at least 24 h [57,58], and butyrate was found to inhibit NFκB activity after 24 and 48 h, but not after 4 and 8 h of incubation in HT-29 cells [56]. This may indicate that the NFκB inhibitory effects of butyrate might set in at a later timepoint in the current model or that pretreatment is required. The CXCL10 release was completely blocked after only 4 h of incubation in IFN-γ+TNF-α-activated IECs, and therefore butyrate blocked the synergistical effect of TNF-α on CXCL10 release independent of NFκB. 

While butyrate did not affect STAT1 phosphorylation, it clearly prevented the induction of IRF9 expression at 4 h and 16 h, and phosphorylation of JAK2 at 16 h in IFN-γ and IFN-γ+TNF-α-activated IECs, therefore blocking the non-canonical STAT1 signaling cascade. At 4 h, butyrate not only prevented the induction of IRF9 expression, but it also prevented transcription of several signaling proteins, such as JAK2. As a consequence, this may have resulted in lower JAK2 protein expression at 16 h of incubation, which would also affect its phosphorylation. The importance of the non-canonical signaling cascade and IRF9 on the expression of CXCL10 in intestinal cells [59] and the influence of HDAC inhibition on IRF9 induction was studied before [51]. Nevertheless, to our knowledge, this is the first study to show that butyrate is capable of having a strong inhibitory effect on IRF9 induction as well and, therefore, we provide novel molecular insights into the anti-inflammatory effect of butyrate as a potent HDAC inhibitor. 

However, the effect of butyrate on other proteins related to the canonical signaling cascade and the non-canonical signaling cascade, such as phosphorylated JAK2, JAK1 and STAT1, has been studied before. Butyrate reduced JAK2 phosphorylation, JAK1 phosphorylation, STAT1 phosphorylation and its nuclear translocation and DNA binding activity in IFN-γ-activated HCT116 or Hke-3 colon carcinoma cell lines. In these cell lines, butyrate affected the canonical signaling cascade, resulting in the prevention of induced CXCL10 release [36,37]. However, phosphorylation of STAT1 and its nuclear translocation was not always observed, for example, in IFN-β-activated lung carcinoma epithelial cells [39]. Even though we did not investigate nuclear translocation of the phosphorylated STAT1 dimer, in contrast to previous studies, STAT1 phosphorylation was not suppressed by butyrate in the current study. Although we confirmed butyrate blocks the non-canonical pathway, we cannot exclude that butyrate blocks the canonical signaling cascade in our model as well. Reduced translocation of phosphorylated STAT1 could result in the accumulation of phosphorylated STAT1 in the cell cytoplasm and we hypothesize that this underlies the inclining pattern of phosphorylated STAT1 protein expression in butyrate treated activated IECs at 16 h of incubation in the current study. Butyrate most prominently inhibits HDAC activity in HT-29 cells, while acetate was least effective. This pattern is similar to the effects of these SCFA on CXCL10 release. Therefore, it was considered that HDAC inhibition plays a role in the mechanism of the effect of SCFAs on the STAT1 signaling cascade. It was observed before that HDAC, and in particular HDAC1, HDAC2 and HDAC3, are required for STAT signaling [48,50]. For example, silencing of HDAC1, HDAC2 and HDAC3 decreased IFN-γ-driven gene activation in Hke-3 cells and overexpression of these HDACs enhanced STAT1-dependent transcription activity [36]. Moreover, a general HDAC inhibitor, similar to SCFAs, also reduced CXCL10 release by IFN-γ-activated human colonic subepithelial myofibroblasts and cervical cancer cells (HeLa cells) and 2fTGH cells [38,48,53]. In the present study, several HDAC inhibitors mimic the effect of SCFAs by inhibiting the induced CXCL10 release in activated IECs. It was not only the general HDAC inhibitor TSA that reduced CXCL10 release, but more specific HDAC inhibitors which inhibit class I and IIb HDAC, did so too, while an HDAC inhibitor which inhibits class IIa HDACs was not effective. Butyrate is known not to inhibit class IIb HDAC [43], so only HDAC from class I could be inhibited by butyrate to prevent induced CXCL10 release. More specific inhibitors of HDAC within class I were studied and all of them inhibited CXCL10 release by IFN-γ-activated IECs, while in IFN-γ+TNF-α-activated IECs, only a class I HDAC1,2,3 inhibitor affected CXCL10 release. Although HDAC inhibitor concentrations used were based on concentrations from previous studies [60,61,62,63,64,65,66,67,68,69,70,71,72], the concentrations used in these studies might not be sufficient to inhibit the strong synergistic effect of IFN-γ+TNF-α. However, higher concentrations could interfere with their selectivity; therefore, we adhered to this concentration range. Overall, our data suggest that butyrate inhibits a combination of HDACs, and that specifically HDAC 1, 2, 3 and 8 (class I) inhibition may have resulted in the suppression of CXCL10 release. HDAC inhibitors mimic the effect of butyrate on induced CXCL10 release; therefore, the effect of either butyrate or TSA on the transcription of genes related to the STAT1 signaling cascade was further investigated. Butyrate indeed prevented IFN-γ and IFN-γ+TNF-α-induced gene expression of *CXCL10*, JAK2, *SOCS1* and *IRF9* in the activated IECs. Butyrate prevented gene transcription in all studied genes at the early timepoint of 4 h, not only of *CXCL10* itself but also of the genes related to its upstream signaling cascade, *JAK2*, *SOCS1* and *IRF9*, which implies gene silencing via an overall blocking mechanism, such as HDAC inhibition. This was further confirmed by the HDAC inhibitor TSA, which showed a similar effect when compared to butyrate. This is in line with previous studies that show the importance of HDAC inhibition in IFN gene suppression [39,48,50]. 

The present study is performed in human colorectal adenocarcinoma cell line HT-29. Although these cells are generally used as an intestinal epithelial model, it would be of additional value to confirm these effects in primary intestinal cells or intestinal biopsies. 

In conclusion, butyrate is the most potent inhibitor of IFN-γ as well as IFN-γ+TNF-α-induced CXCL10 release of activated IECs when compared to propionate and acetate. Butyrate already completely blocks CXCL10 release at 2 mM. Butyrate blocks CXCL10 release via HDAC inhibition and its efficacy is comparable to known HDAC inhibitors, such as TSA. The HDAC inhibition causes downregulation of the expression of genes and proteins related to the non-canonical STAT1 pathway, including IRF9 and CXCL10 itself, resulting in the prevention of induced CXCL10 release. HDAC inhibitors are getting more and more attention as potential therapeutic agents because of their anti-inflammatory properties [73]. Our findings show that butyrate is a very effective HDAC inhibitor, which demonstrates the importance of assuring sufficient butyrate levels in the intestine by means of diet or, perhaps, by supplementation or pharmaceutical therapy. The essential role for butyrate in controlling homeostasis and maintaining gut health has been shown in experimental models, while in addition, butyrate may contribute to the prevention of systemic diseases via its immunoregulatory effects [74,75,76,77]. The current manuscript further highlights the great potential of butyrate to act as a controller of intestinal homeostasis by blocking unwanted epithelial activation, which may contribute to lowering the risk of local and systemic inflammation, and which are both understood to contribute to development of non-communicable diseases. 

## 4. Materials and Methods

### 4.1. Intestinal Epithelial Cell Culture 

Human IECs, HT-29 cells (ATCC, HTB-38, Manassas, VA, USA; passages 150–175) were grown in McCoy’s 5A medium (Gibco, Invitrogen, Carlsbad, CA, USA) supplemented with 10% heat-inactivated fetal calf serum and 1% penicillin/streptomycin (100 U/mL and 100 ug/mL) (Sigma-Aldrich, St. Louis, MO, USA). The cells were cultured in 75 cm^2^ flasks (Greiner Bio-One, Alphen aan den Rijn, The Netherlands) and passaged once a week. Cells were kept in an incubator at 37 °C in the presence of 5% CO_2_.

### 4.2. Epithelial Activation and SCFAs and HDAC Inhibitor Treatment

HT-29 were seeded in 48 or 96 well plates (Corning, New York, NY, USA) and grown till confluency before they were used to perform experiments. The medium was changed every 2–3 days and 1 day prior to the experiment. After reaching confluence, cells were activated with IFN-γ (100 IU/mL) or IFN-γ (100 IU/mL) + TNF-α (1 ng/mL) (Invitrogen) and simultaneously treated with one of the SCFAs or HDAC inhibitors for 1, 4, 16 or 24 h. As SCFAs, either sodium butyrate (2, 4, 8 mM), sodium propionate (2, 4, 8 mM) or sodium acetate (4, 8, 16 mM) (Sigma-Aldrich) were used dissolved in culture medium, and as HDAC inhibitors, either Trichostatin A (TSA) (10, 100 μM), Tinostamustine (1, 5, 15 μM), Tacedinaline (1, 10 μM), Droxinostat (10, 50 μM), RGFP966 (5, 20 μM), Cay10683 (5, 20 μM) or TMP269 (10, 20, 50 μM) (MedChemExpress, Sollentuna, Sweden) were used dissolved in DMSO and further diluted in culture medium. Table 1 indicates the HDAC inhibitor specificity. A quantity of 0.5% DMSO in culture medium served as a control. After 1, 4, 16 or 24 h of incubation, supernatants were collected for enzyme-linked immunosorbent assay (ELISA) and cells were either lysed in LBP lysis buffer (Macherey-Nagel, Düren, Germany) for quantitative polymerase chain reaction (qPCR) analysis or in RIPA lysis buffer (Thermo Fisher Scientific, Waltham, MA, USA), supplemented with protease and phosphatase inhibitors (Roche, Diagnostics, Mannheim, Germany) for Western blot analysis. At 24 h, the viability of the cells was assessed.

### 4.3. HDAC Activity Assay

To determine the effect of SCFAs on HDAC activity in HT-29 cells, an HDAC activity assay (In Situ Histone Deacetylase Activity Fluorometric Assay Kit, Sigma-Aldrich) was performed according to the manufacturer’s protocol. In short, cells were grown in white clear bottom 96 well plates. After reaching confluence, cells were treated with either sodium butyrate (2, 4 mM), sodium propionate (2, 4 mM) or sodium acetate (4, 8 mM). HDAC inhibitor TSA (10 μM) served as positive controls. After 24 h substrate was added, and cells were incubated for 2 h. Subsequently, developer mix was added, and cells were incubated for 30 min, after which fluorescence was read at Excitation/Emission 368/442 nm in a microplate reader (GloMax Discover, Promega Corporation, Madison, WI, USA).

### 4.4. ELISA

CXCL10 and CXCL8 secretion was measured in the supernatant with the use of a commercially available ELISA kit (CXCL10: BioLegend, San Diego, CA, USA or R&D Systems, Minneapolis, MN, USA; CXCL8: Invitrogen, Thermo Fisher Scientific) both according to the manufacturer’s protocol. In short, high-binding 96-well plates were coated with capture antibody and incubated overnight. Non-specific binding was blocked for 1 h at room temperature. After washing, the samples or the standard were added for 2 h at room temperature. Then, plates were washed and incubated with streptavidin-horseradish peroxidase for 1 h at room temperature. Subsequently, the plates were washed and incubated in the dark with substrate solution at room temperature. The reaction was stopped with 1 M H_2_SO_4_ and absorbance was measured at 450 nm in a microplate reader (iMark, Bio-Rad Laboratories, Hercules, CA, USA or GloMax Discover, Promega Corporation, Madison, WI, USA).

### 4.5. Western Blot

First, protein concentration was determined using a BCA protein assay kit (Thermo fisher Scientific) to ensure equal protein loading across samples. Bromophenol blue and 1,4-Dithiothreitol were added to the samples to denature the proteins. Protein samples were then added to a Criterion^TM^ 4–20% Tris-HCl gel (Bio-Rad, Veenendaal, The Netherlands) for separation with electrophoresis. Thereafter the proteins were transferred to a polyvinylidene difluoride membrane (Transblot Turbo, Bio-Rad). The membrane was blocked using 5% milk protein in phosphate-buffered saline containing 0.05% Tween-20. After blocking, the membrane was incubated with primary antibodies overnight at 4 °C. As primary antibodies phosphorylated JAK2 (1:200, Thermo Fisher Scientific), phosphorylated STAT1 (1:200, R&D systems), phosphorylated NF-kappaB p65 (1:1000, Cell signaling, Danvers, MA, USA), IRF9 (1:1000, Cell signaling) and β-actin (1:1000, Cell signaling) were used. After incubation, the membranes were washed and incubated with horseradish peroxidase-conjugated secondary antibodies (Dako, Santa Clara, CA, USA) for 2 h. Membranes were again washed and the proteins on the membranes were visualized using ECL reagent (Bio-Rad) for phosphorylated NF-kappaB p65 and β-actin or ECL^TM^ Prime (Cytiva, Marlborough, MA, USA) for the other proteins of interest that were assessed. Data was analyzed using Image J (Wayne Rasband, National Institutes of Health, Bethesda, MD, USA). When the same membrane was used for the analysis of multiple proteins with different sizes, the membrane was stripped using Restore PLUS Western Blot Stripping Buffer (Thermo Fisher Scientific). 

### 4.6. qPCR

First, RNA was isolated from lysed cell homogenates using a NucleoSpin^®^ RNA Plus kit (Macherey-Nagel) according to the manufacturer’s protocol. DNAse (Qiagen, Hilden, Germany) was used to remove contaminated DNA. Second, complementary DNA was synthesized using an iScript^TM^ cDNA synthesis kit (Bio-Rad) according to the manufacturer’s protocol. Quantitative analysis was performed on a CFX96 real-time PCR detection system with the use of IQ^TM^ SYBR^®^ Green Supermix (both from Bio-Rad). Commercially available primers for SOCS1, JAK2, CXCL10 and IRF9 were obtained as genes of interest and ribosomal protein S13 (RPS13) was obtained as a reference gene (all from Qiagen). Relative mRNA expression was calculated as 100 × 2[Ct reference–Ct gene of interest] [78]. 

### 4.7. Viability Assay

After 24 h, cell viability was determined using a WST-1 assay (Roche) according to the manufacturer’s protocol. In short, WST was diluted in culture medium (1:10 dilution) and added to each well for 30 min at 37 °C in the presence of 5% CO_2_. Subsequently, 100 μL of each well was transferred to a clear 96 well plate and absorbance measured at 450 nm with a microplate reader (iMark, Bio-Rad Laboratories, Hercules, CA, USA or GloMax Discover, Promega Corporation, Madison, WI, USA).

### 4.8. Statistical Analysis

The data is expressed as mean ± standard error of the mean (SEM) of 3 or 4 independent measurements. All statistical analyses were performed using GraphPad Prism software version 8.4.3. (GraphPad Software, San Diego, CA, USA). Data was normally distributed and statistical significance was tested using the repeated measures one-way ANOVA analysis, followed by Bonferroni’s post hoc test with selected pairs or, if indicated, with a paired two-sample *t*-test. Results were considered statistically significant when *p* < 0.05.

## Figures and Tables

**Table 1:** HDAC inhibition by specific HDAC inhibitors, categorized per class. Intensity in color indicates half-maximal inhibitory concentration of the HDAC inhibitor in mM, μM, nM or pM range (light grey to black).

				Class I				Class IIb		Class IIa			
HDAC				1	2	3	8	6	10	4	5	7	9
TSA													
Butyrate													
TMP269													
Tinostamustine													
Tacedinaline													
Droxinostat													
RGFP966													
Cay10683													
mM	μM	nM	pM										

**Figure A1:**
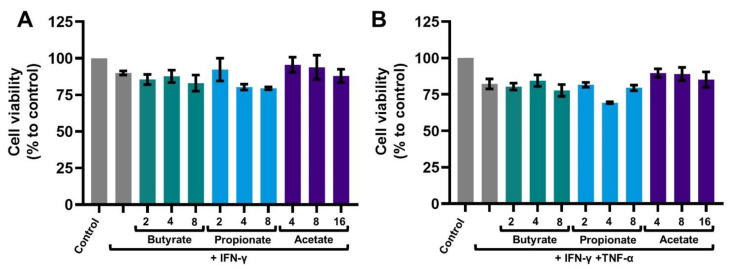
Viability of intestinal epithelial cells after 24 h, activated with IFN-γ (**A**) or IFN-γ+TNF-α (**B**) and treated with 2, 4, 8 and 16 mM butyrate, propionate or acetate. Data are represented as mean ± SEM (*n* = 3).

## Data Availability

The data presented in this study are available on request from the corresponding author.

## Abbreviations

SCFAs	Short Chain Fatty Acids
IFN-γ	Interferon-gamma
TNF-α	tumor necrosis factor-alpha
IECs	Intestinal epithelial cells
STAT1	transducer and activator of transcription 1
HDAC	histone deacetylase
JAK1	Janus kinase 1
JAK2	Janus kinase 2
IRF9	Interferon Regulatory Factor 9
CXCL10	C-X-C motif chemokine ligand 10
qPCR	quantitative polymerase chain reaction
ELISA	Enzyme-Linked Immunosorbent Assay
SOCS1	suppressor of cytokine signaling 1
RPS13	ribosomal protein S13
CXCL8	C-X-C motif chemokine ligand 8
NFκB	Nuclear Factor kappa-light-chain-enhancer of activated B cells
TSA	Trichostatin A
